# Efficacy of Various Antidiabetic Agents as Add-On Treatments to Metformin in Type 2 Diabetes Mellitus: Systematic Review and Meta-Analysis

**DOI:** 10.5402/2012/798146

**Published:** 2012-03-27

**Authors:** Nalinee Poolsup, Naeti Suksomboon, Wanwaree Setwiwattanakul

**Affiliations:** ^1^Department of Pharmacy, Faculty of Pharmacy, Silpakorn University, Nakhon-Pathom 73000, Thailand; ^2^Department of Pharmacy, Faculty of Pharmacy, Mahidol University, Bangkok 10400, Thailand

## Abstract

*Background and Aim*. Diabetes mellitus is a chronic disease that has a great impact on patients and society. Metformin monotherapy is capable of maintaining a target glycemic control only for a short term. The aim of this study was to determine the efficacy of combination therapy of metformin with any antidiabetic agents in type 2 diabetes mellitus (T2DM) patients. *Methods*. Reports of randomized controlled trials (RCTs) of combination therapy of metformin with various antidiabetic agents in T2DM failing metformin alone were identified. *Results*. Eight studies were identified in our paper. Thiazolidinediones (TZDs) were as effective as dipeptidyl peptidase IV inhibitors (DPP IV inhs) in reducing HbA1c value (pooled mean difference −0.03%; 95% CI −0.16 to 0.10%). In comparison between TZDs and sulphonylureas (SUs), TZDs reduced fasting plasma insulin (FPI) more effectively than SUs (pool mean difference −5.72 *μ*U/mL; 95% CI −8.21 to −3.22 *μ*U/mL, *P* < 0.00001), but no significant differences were detected in the effects on HbA1c and fasting plasma glucose (FPG) (pooled mean difference −2.19 mg/dL; 95% CI −11.32 to 6.94 mg/dL, *P* = 0.64). *Conclusions*. Our study showed that TZDs reduced FPG better than did DPP IV inhs and decreased FPI more than did SUs.

## 1. Introduction

Diabetes mellitus is a chronic disease that has a great impact on patients and society. The estimate of worldwide prevalence by the International Diabetes Federation (IDF) in 2007 is about 246 million people, most of whom (85–95%) have type 2 diabetes mellitus (T2DM) [1]. The complications are the major causes of morbidity and mortality of people with diabetes [2–4]. Antidiabetic agents have an important role in normalizing plasma glucose levels [5, 6]. Metformin is now recommended as the first agent for blood glucose lowering in type 2 diabetes patients [7–11]. Metformin has been proven to be efficacious in reducing cardiovascular risk [12] and is the only pharmacological treatment that could improve macrovascular outcomes in patients with diabetes [8]. However, metformin monotherapy is capable of maintaining a target glycemic control only for a short term [13]. Combination therapies of metformin with other oral antidiabetic agents are therefore necessary. There are many therapeutic options of adding second agents in metformin-treated subjects which are recommended [7–11], such as insulin, insulin secretagogues, thiazolidinediones (TZDs), glucagon-like-peptide-1 (GLP-1) analogues, and dipeptidyl peptidase IV inhibitors (DPP IV inhs).

Our paper was aimed at determining the efficacy of combination therapy of metformin with any antidiabetic agents in type 2 diabetes inadequately controlled with metformin alone.

## 2. Methods

### 2.1. Search Strategy

 Reports of randomized controlled trials of combination therapy of metformin with various antidiabetic agents in type 2 diabetes failing metformin monotherapy were identified through a systematic literature search of MEDLINE (Pubmed), EMBASE, and The Cochrane Library. The following MeSH terms were used: diabetes mellitus type 2, metformin, sulfonylurea compounds, thiazolidinediones, dipeptidyl peptidase IV inhibitors, insulin, insulin NPH, and insulin long acting. This was followed by keyword search using as keywords glibenclamide, glyburide, gliclazide, glimepiride, glipizide, chlorpropamide, tolbutamide, meglitinide, nateglinide, repaglinide, pioglitazone, rosiglitazone, sitagliptin, vildagliptin, saxagliptin, alogliptin, glargine, lispro, aspart, glulisine, detemir, acarbose, voglibose, miglitol, exenatide, liraglutide, and pramlintide. Historical searches of reference lists of relevant randomized controlled trials, systematic and narrative reviews were also undertaken. No language restriction was imposed.

### 2.2. Study Selection

Eligible studies were selected by two reviewers, and differences were resolved by agreement. The studies were included in this systematic review, if they (a) were randomized controlled trials in type 2 diabetes patients who had already been treated with metformin alone, (b) compared between two different antidiabetic drugs in combination with metformin, (c) included patients with baseline HbA1c ≥ 7%, (d) had no addition of a third oral antidiabetic agent or insulin, (e) lasted at least 12 weeks of treatment duration, and (f) reported outcome measure in terms of hemoglobin A1c (HbA1c). Abstract presentations were excluded.

We selected 12 weeks as the minimal study duration to assure the effect of medication on HbA1c level. HbA1c at ≥7% was chosen because the recent meta-analysis of the benefit of intensive glucose-lowering treatment reported overall HbA1c target level of <7%. Intensive glycemic control group (HbA1c < 7%) had no significant effects on stroke and all-cause mortality when compared with standard treatment where HbA1c goal was ≥7% [14]. Some guidelines recommend a target of HbA1c at ≤6.5% [7, 8, 11]. Achieving these goals may prove difficult. Only 60% of subjects have been reported to reach an HbA1c goal of ≤7.5% [15]. In addition, the ADA/EASD consensus treatment algorithm for the metabolic management of diabetes recommends a HbA1c goal at <7% [9, 10].

### 2.3. Data Extraction and Quality Assessment

Data extraction and study quality assessment were performed independently by two investigators using a standardized form. Disagreements were resolved by a third investigator. The data extracted were publication year, study country, study design, study duration, outcome parameters, type and dosage of interventions, patient characteristics, and number of participants.

 The methodological quality of each trial was assessed using the scale developed by Jadad et al. [16]. The Jadad's scale is divided into three dimensions: randomization, blinding and reasons for withdrawals and dropouts. The possible maximum score is 5 points, the studies with the score of 2 points or less are of low quality, while those with 3 points or more are of high quality.

### 2.4. Statistical Analysis

Primary outcome was HbA1c, secondary outcomes were fasting plasma glucose (FPG) and fasting plasma insulin (FPI). Efficacy was reported as mean change value from baseline to final assessment. When the variations of these changes were not reported, we estimated them by using the following equation: [17]

(1) $$\text{S}{\text{D}}_{1}\left(C\right)=\sqrt{\text{S}{\text{D}}_{1}{\left(B\right)}^{2}+\text{S}{\text{D}}_{1}{\left(F\right)}^{2}-\left(2R\times \text{S}{\text{D}}_{1}\left(B\right)\times \text{S}{\text{D}}_{1}\left(F\right)\right)},$$

where SD_1_(*C*) is the standard deviation of change, SD_1_(*B*) and SD_1_(*F*) are the standard deviations of baseline and final values: respectively, and *R* is the correlation coefficient and was assumed to be 0.5. In addition, when the variations were not reported at all, the pooled SD calculated from the studies data that reported SD was used.

Treatment effect was estimated with mean difference in the change value between the treatment group and the control group. The inverse variance-weighted method was used for the pooling of mean difference and the estimation of 95% confidence interval [18]. Random effects model was used to combine the results of individual studies when Q-statistic test was significant at the level of 0.1 [19], otherwise the fixed effects model was used [18]. I-squared statistic which is the percentage of total variation across studies was used to quantify the level of heterogeneity [20]. Sensitivity analysis was performed to examine the causes of heterogeneity. Publication bias was assessed by the method of Egger et al. [21]. The statistical analysis was undertaken with RevMan 5.0 (Cochrane collaboration). The statistical significance was set at *P* < 0.05.

## 3. Results

### 3.1. Study Characteristics

 The initial search identified 121 potentially relevant randomized studies of additional therapy to metformin in type 2 diabetes mellitus. All were published in English. One hundred and thirteen trials were excluded for the following reasons. Seven trials were excluded as they evaluated agents already withdraw from the market, that is, inhaled insulin, muraglitazar, and tesaglitazar. Forty-five trials that included subjects previously receiving various antidiabetic regimens including metformin and did not report data separately for each antidiabetic agent therefore were also excluded. Thirty-three studies were excluded since they were placebo-controlled or non-treatment-controlled. Nine trials with entry HbA1c < 7% were excluded. We excluded one trial which added a third oral glucose-lowering agent or insulin. In addition, this particular trial was previously reported in three preliminary publications and therefore was excluded. One trial was excluded as the duration of study was less than 12 weeks. Seven trials were further excluded because they were duplication or interim analysis. One trial evaluated vildagliptin 100 mg once daily which is currently not a recommended dose (the recommended dose is now 50 mg twice daily), thus it was excluded. Six studies were abstract presentation and were then excluded. The remaining eight trials met our inclusion criteria and were included in the meta-analysis [22–29].

Of the eight trials, two compared thiazolidinediones (TZDs) versus dipeptidyl peptidase IV inhibitors (DPP IV inhs) [22, 23], four assessed TZDs against sulphonylureas (SUs) [24–27], the rest evaluated pioglitazone versus rosiglitazone [28] and biphasic insulin aspart 30 versus glibenclamide [29]. Characteristics of these studies are presented in Table 1.

### 3.2. Efficacy

#### 3.2.1. TZDs versus DPP IV Inhs

**Figure 1:**
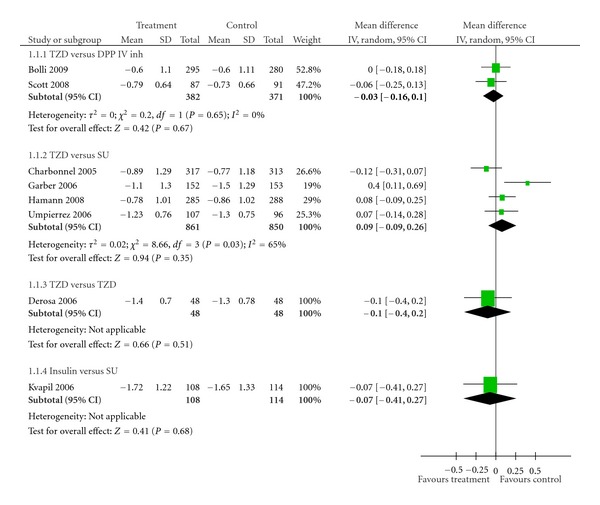
Effects of various antidiabetic agents on HbA1c as an add-on treatment to metformin in T2DM.

**Figure 2:**
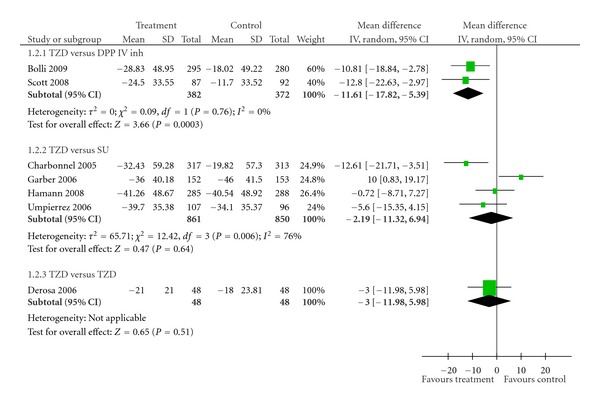
Effects of various antidiabetic agents on FPG as an add-on treatment to metformin in T2DM.

**Figure 3:**
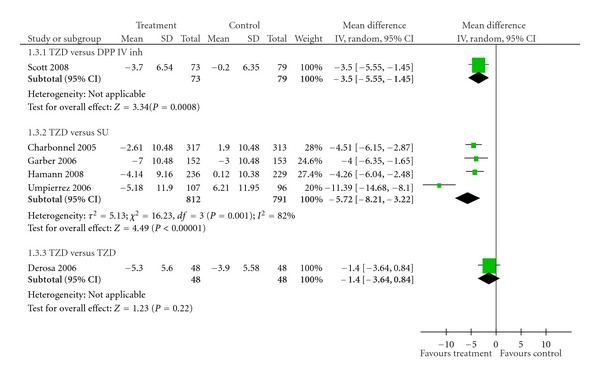
Effects of various antidiabetic agents on FPI as an add-on treatment to metformin in T2DM.

There were a total of 753 T2DM patients treated with metformin in the two trials that compared TZDs against DPP IV inhs [22, 23]. No significant difference in effect on HbA1c was observed between TZDs and DPP IV inhs (pooled mean difference −0.03%; 95% CI −0.16 to 0.10%) (Table 2, Figure 1). However, TZDs induced a greater reduction of FPG than did DPP IV inhs (pooled mean difference −11.61 mg/dL; 95% CI −17.82 to −5.39 mg/dL, *P* = 0.0003) (Table 3, Figure 2). Only one trial reported FPI data [22]. FPI significantly decreased with TZDs compared with DPP IV inhs (mean difference −3.50 *μ*U/mL; 95% CI −5.55 to −1.45 *μ*U/mL, *P* = 0.0008) (Table 4, Figure 3).

#### 3.2.2. TZDs versus SUs

**Figure 4:**
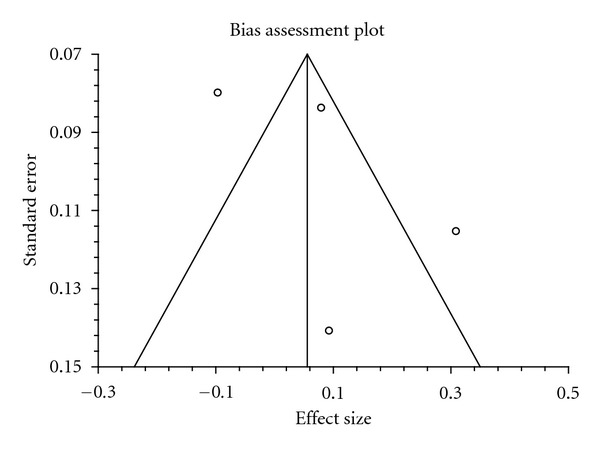
Funnel plot of the studies included in the comparison of TZDs versus SUs.

A total of 1,711 subjects in four studies received TZDs or SUs as an add-on to metformin [24–27]. TZDs were no more effective than SUs in decreasing HbA1c (pooled mean difference 0.09%; 95% CI −0.09 to 0.26%) (Table 2, Figure 1). In addition, the same result was observed among the FPG values. The effect of TZDs in improving FPG was no better than that of SUs (pooled mean difference −2.19 mg/dL; 95% CI −11.32 to 6.94 mg/dL, *P* = 0.64) (Table 3, Figure 2). On the other hand, TZDs decreased FPI greater than did SUs (pool mean difference −5.72 *μ*U/mL; 95% CI −8.21 to −3.22 *μ*U/mL, *P* < 0.00001) (Table 4, Figure 3). No publication bias was detected in comparing TZDs with SUs in terms of HbA1c (Egger bias 4.17; 95% CI −11.82 to 20.16, *P* = 0.38) (Figure 4).

#### 3.2.3. TZDs versus TZDs

One trial that compared pioglitazone versus rosiglitazone and involved 96 subjects [28] showed no differences in effects on HbA1c (mean difference −0.10%; 95% CI −0.40 to 0.20%) (Table 2, Figure 1), FPG (mean difference −3.00 mg/dL; 95% CI −11.98 to 5.98 mg/dL) (Table 3, Figure 2), and FPI (mean difference –1.40 *μ*U/mL; 95% CI −3.64 to 0.84 *μ*U/mL) (Table 4, Figure 3).

#### 3.2.4. Insulin versus SUs

 One study examined the effect of biphasic insulin aspart 30 (*n* = 108) against glibenclamide (*n* = 114) [29]. There was no significant difference in effect on HbA1c between the two groups (mean difference −0.07%; 95% CI −0.41 to 0.27%).

## 4. Discussion

 The results of this analysis suggest that TZDs were as effective as DPP IV inhs in reducing HbA1c value in type 2 diabetes patients who had been treated with metformin alone, however, FPG better improved with TZDs than with DPP IV inhs. From its mechanism of actions, TZDs may reduce FPI more than does DPP IV inhs. In addition, there are some issues that should be concerned. First, patients in both trials received fixed-dose TZDs (rosiglitazone 8 mg/day [22], pioglitazone 30 mg/day [23]) and there was no re-titration. Indeed, dose of TZDs in combination therapy with metformin should be titrated from initial dose to the maximum dose (rosiglitazone 4–8 mg/day, pioglitazone 15–45 mg/day) based on efficacy in reducing hyperglycaemia and tolerability such as side effects. Second, maximum dose of TZD was not used (pioglitazone 30 mg OD was used) whiles maximum recommended dose of the DPP IV inh (vildagliptin 50 mg bid) was used in one trial and patients compliance was not determined [23]. That may affect the comparability between the comparators. 

TZDs reduced FPI more effectively than SUs, but no significant differences were detected in the effects on HbA1c and FPG. All subjects in the included studies had hyperinsulinemia (Table 4). Hyperinsulinemia is recognized as a risk factor that affects the development and progression of atherosclerosis and cardiovascular disease which are the major causes of morbidity and mortality in diabetes patients [30]. TZDs improve glycaemic control by promoting the local effect of insulin. TZDs also decrease the gluconeogenesis from hepatic tissues, resulting in reduced insulin resistance, which leads to improved glycemic control with no enhancement in the insulin secretion. [31], while SUs improve blood glucose level by triggering insulin release from the pancreatic *β*-cell [32]. Thus, TZDs and SUs may have similar effect in controlling blood glucose level but have different effect on FPI level. 

 Evidence of interstudy heterogeneity was discovered in the comparison of TZDs against SUs based on HbA1c (*χ* ^2^ = 8.66, *P* = 0.03; *I* ^2^ = 65%, Figure 1). Similar results were found with FPG outcome (*χ* ^2^ = 12.42, *P* = 0.006; *I* ^2^ = 76%) and FPI parameter (*χ* ^2^ = 16.23, *P* = 0.001; *I* ^2^ = 82%). Sensitivity analysis was performed by excluding Garber's study [25] which was different from other studies in various aspects. Firstly, it lasted only 24 weeks, the shortest treatment duration among the trials that compared TZDs against SUs. Hence, TZD may not demonstrate maximum effectiveness in reducing hyperglycemia that was supported by ADOPT trial in which TZDs showed the maximum benefit in decreasing HbA1c at 48 week [33]. Secondly, the dose of metformin could be up- and downtitrated, while other trials used metformin at baseline dose and maintained the dose throughout the course of studies. Finally, the medication could be titrated at every visit (4 week thereafter for 24 weeks) depending on mean daily glucose or fructosamine levels, while other trials employed restriction of forced-titration period (approximately 1 to 16 weeks). When this study was excluded, no significant heterogeneity was observed with regard to HbA1c (*χ* ^2^ = 2.74, *P* = 0.25; *I* ^2^ = 27%) and FPG results (*χ* ^2^ = 3.70, *P* = 0.16; *I* ^2^ = 46%). The effect estimates remained unchanged. The pooled mean differences were 0.01% (95% CI −0.11 to 0.14%) and −6.06 mg/dL (95% CI −13.08 to 0.95 mg/dL), respectively for HbA1c and FPG values. Interstudy heterogeneity in the pooling of FPI may be attributable to the study by Umpierrez G et al. 2006 [26]. In this study, the patients in the SU group had higher body mass index (BMI) (mean 34.5 kg/m^2^) compared with those reported in other studies (mean 32.5 kg/m^2^). Excess body weight was shown to be directly correlated with hyperinsulinemia [33], thus reduced effect on FPI in the SU group. Sensitivity analysis conducted by excluding this trial revealed no significant heterogeneity (*χ* ^2^ = 0.13, *P* = 0.94; *I* ^2^ = 0%). The effect of TZDs remained unaffected. FPI significantly reduced with TZDs compared with SUs (pooled mean difference −4.31 *μ*U/mL; 95% CI −5.39 to −3.24 *μ*U/mL, *P* < 0.00001). 

We found that our results contrasted with those previously reported by Monami et al. 2008 [34] where SUs demonstrated a greater efficacy in reducing HbA1c compared with TZDs. Monami results were based on clinical trials up to and including January 2007. 

There were variations and limitations among the included studies. Several treatment strategies including diet control, exercise training, and antidiabetic agents are useful tools to improve glycaemic control in diabetes. Only two studies provided details about dietary advice [24, 25]. There were variations in criteria for dose titration. Garber et al. [25] titrated study medication according to mean daily glucose levels measured by self-monitoring of blood glucose (SMBG) method or fructosamine levels. In addition, downward titration was permitted only in glibenclamide-arm [25]. While in other trials, the study medications were titrated to higher or lower doses based on tolerability, such as actual hypoglycemia or enhanced risk of hypoglycemia [24]. Umpierrez et al. [26] administered active agent with forced titration to a maximum dose or adjusted dose based on fasting SMBG. However, only pioglitazone treatment was uptitrated based on HbA1c levels [26]. Down- and uptitrating the dose were also performed according to mean daily glucose from SMBG results [27]. Furthermore, the duration allowed for the dose titration also varied among the included trials. Most trials had limited period for dose titration and maintained that dose until the end of study except the study by Garber et al. in which the dose could be increased or decreased every 4 weeks throughout 24-week study duration [25]. In the other trial, duration for dose titration differed between the intervention and the control groups [26]. 

The methodological quality of the included studies was assessed by Jadad's score. Studies scoring 3 or greater were rated as high quality trials [16]. Of the eight trials, seven [22–25, 27–29] were rated as high quality. One study was not double-blind study and did not report the method to generate allocation and the reasons for dropout [26].

A statistical pooling of pioglitazone versus rosiglitazones and biphasic insulin aspart 30 versus glibenclamide was not possible. However, in both trials, there were no significant differences between the treatment and the control groups in all outcomes. This may be explained by the fact that pioglitazone and rosiglitazone are categorized in the same glucose-lowering agent group. In comparison between biphasic insulin aspart 30 and glibenclamide, the initial dose of biphasic insulin aspart 30 was 0.2 U/kg/day and the dose was titrated every one to seven days in step of two to four units per injection. Thus, patients whose blood glucose levels were uncontrolled with suggested dose and required additional dose were absolutely not able to achieve target glycaemic goals. 

## 5. Conclusions

 TZDs, DPP IV inhs, and SUs may have similar effect in reducing HbA1c in type 2 diabetes patients whose blood glucose levels were not adequately controlled with metformin alone. However, TZDs decreased FPG better than did DPP IV inhs and reduced FPI more than did SUs. Given the limitations of the published data, large sample size, high quality, randomized controlled trials of combination treatment with metformin, and other agents are warranted. 

## Figures and Tables

**Table 1:** Characteristics of the studies included in the meta-analysis.

Study, study origin	Quality score	Inclusion criteria	Design and duration (week)	Intervention/day	*n*
*TZDs versus DPP IV inhs.*					

(i) Scott et al. [22] Australia, India, Italy, Malaysia, New Zealand, Poland, Sweden	3	(i) T2DM (ii) A1c 7–11% (iii) Taking metformin monotherapy ≥1,500 mg/day for ≥10 weeks (iv) Aged 18–75 years	DP (18)	(i) Rosiglitazone 8 mg OD + metformin (≥1,500 mg/day) usual dose (ii) Sitagliptin 100 mg OD + metformin (≥1,500 mg/day) usual dose	(i) 87 (ii) 94

(ii) Bolli et al. [23] Australia, Austria, Germany, Italy, UK, USA, South Africa, Spain	3	(i) T2DM (ii) A1c 7.5–11.0% (iii) Receiving metformin alone ≥1,500 mg/day (iv) Aged 18–77 years (v) Male and female (non-fertile or of childbearing potential using a medically approved birth control method) (vi) BMI 22–45 kg/m^2^ (vii) FPG < 15 mmol/L	DP (52)	(i) Pioglitazone 30 mg OD + metformin (≥1,500 mg/day) usual dose (a) mean metformin dose 2,020 mg (ii) Vildagliptin 50 mg bid + metformin (≥1,500 mg/day) usual dose (a) mean metformin dose 2,020 mg	(i) 281 (ii) 295

*TZDs versus SUs*					

(i) Charbonnel et al. [24] 29 European countries, Australia, Canada, South Africa	5	(i) T2DM (ii) A1c 7.5–11.0% (iii) Managed with metformin monotherapy (≥50% of the maximum recommended dose or maximum tolerated dose) for ≥12 weeks (iv) Aged 35–75 years (v) Fasting C-peptide levels ≥0.50 nmol/L (1.5 ng/mL) (vi) Stable or worsening glycemic control for ≥3 months	DP (104)	(i) Pioglitazone 15–45 mg (titrated) + metformin (>50% maximum dose or max-tolerated dose) usual dose (a) Mean metformin dose 1,726 mg (b) Mean pioglitazone dose 39 mg (ii) Gliclazide 80–320 mg + metformin (>50% maximum dose or max-tolerated dose) usual dose (a) Mean metformin dose 1,705 mg (b) Mean gliclazide 212 mg	(i) 317 (ii) 313

(ii) Garber et al. [25] USA	4	(i) T2DM (ii) A1c > 7.0 and ≤12.0% (iii) On metformin monotherapy ≥1,500 mg/day for ≥8 weeks (iv) Aged 20–78 years (v) BMI 23–45 kg/m^2^ (vi) Willing and able to perform SMBG (vii) Female of childbearing potential had to practise acceptable methods of birth control and to have negative pregnancy test results within 72 hours of study treatment	DP (24)	(i) Used metformin 1,500 mg: metformin 1,500–2,000 mg (titrated) + rosiglitazone 4–8 mg (titrated) (ii) Used metformin >1,500 mg: metformin 2000 mg + rosiglitazone 4–8 mg (titrated) (a) Mean final metformin/rosiglitazone dose 1,819/7.1 mg (iii) Metformin-glibenclamide 1,000–2,000/5–10 mg (a) Mean final metformin/glibenclamide dose 1,509/7.6 mg	(i) 158 (ii) 160

(iii) Umpierrez et al. [26] USA	2	(i) Diagnosed of T2DM at least 6 months (ii) A1c 7.5–10% (iii) Treated with metformin (1–2.5 g/d) or extended-release metformin alone (0.5–2.0 g/d) for ≥8 weeks (iv) Aged 18–79 years (v) BMI ≥ 24 kg/m^2^ (vi) FPG 126–235 mg/dL (vii) Fasting C-peptide ≥ 0.27 nmol/L	OP (26)	(i) Pioglitazone 30–45 mg (titrated) + metformin usual dose (a) Mean final metformin dose 1,570 mg (ii) Glimepiride 2–8 mg (titrated) + metformin usual dose (a) Mean final metformin dose 1,490 mg	(i) 109 (ii) 101

(iv) Hamann et al. [27] Europe, Mexico	4	(i) Male and female with T2DM (ii) A1c 7–10% (iii) Having received metformin (≥0.85 g/day) for ≥8 weeks (iv) BMI ≥ 25 kg/m^2^	DP (52)	(i) Rosiglitazone 4–8 mg (titrated) + metformin 2,000 mg (a) Mean final dose of rosiglitazone/metformin 7.7/2,000 mg (ii) Glibenclamide 5–15 mg (titrated) + metformin 2,000 mg (iii) Gliclazide 80–320 mg (titrated) + metformin 2,000 mg (a) Mean final dose of glibenclamide/metformin 11/2,000 mg (b) Mean final dose of gliclazide/metformin 238.1/2,000 mg	(i) 294 (ii) 302

*TZD versus TZD*					

(i) Derosa et al. [28] Italy	5	(i) T2DM duration ≥6 months (ii) A1c > 7.5% or had adverse effects with diet and metformin (administered up to the maximum tolerated dose) (iii) Caucasian patients aged ≥18 years (iv) BMI 25.0–28.1 kg/m^2^ (v) Diagnosed with metabolic syndrome according to the NCEP Treatment Panel III (vi) TG ≥ 150 mg/dL (vii) Hypertension according to the WHO criteria (blood pressure, ≥130/≥85 mmHg) (viii) Fasting C-peptide > 1.0 ng/mL	DP (48)	(i) Pioglitazone 15 mg + metformin 1,500–3,000 mg (titrated) (a) Mean metformin dose 2,250 ± 750 mg (ii) Rosiglitazone 4 mg OD + metformin 1,500–3,000 mg (titrated) (a) Mean metformin dose 2,250 ± 750 mg	(i) 48 (ii) 48

*Insulin versus SU*					

(i) Kvapil et al. [29] Croatia, Czech Republic, Denmark, France, Greece, Hungary, Norway, Poland, Portugal, Russia, Spain	3	(i) T2DM (ii) Receiving ≥850 mg metformin monotherapy for ≥4 weeks	OP (16)	(i) BIAsp 30 was 0.2 U/kg body weight (could be titrated) + metformin (maximum tolerated or maximum effective dose, titrated) (a) Mean metformin dose 1,660 mg (ii) Glibenclamide 1.75–10.5 mg (titrated) + Metformin (maximum tolerated or maximum effective doses, titrated) (a) Mean metformin dose 1,660 mg	(i) 116 (ii) 114

DP: double blind parallel, OP: open label parallel, BIAsp 30: biphasic insulin aspart 30, T2DM: type 2 diabetes mellitus, T1DM: type 1 diabetes mellitus, TZDs: thiazolidinediones, Clcr: creatinine clearance, BMI: body mass index, FPG: fasting plasma glucose, SUs: sulphonylureas, Hgb: hemoglobin, SMBG: self-monitoring blood glucose, WHO: World Health Organization, NCEP: National Cholesterol Education Program.

**Table 2:** Summary of HbA1c (%) between the treatment and the control groups.

Study	Treatment				Control				Difference
	*n*	Baseline	Final	Change	*n*	Baseline	Final	Change	between groups
				*TZDs versus DPP IV inhs.*					

(i) Scott et al. [22] Rosiglitazone versus Sitagliptin	87	7.73 ± 0.81	6.94 ± 0.75	−0.79 ± 0.64	91	7.75 ± 0.99	7.01 ± 0.86	−0.73 ± 0.66	−0.06
(ii) Bolli et al. [23] Pioglitazone versus Vildagliptin	295	8.48 ± 0.86 (SE = 0.05)	7.64 ± 1.89 (SE = 0.11)	−0.6 ± 1.1	280	8.4 ± 0.84 (SE = 0.05)	7.73 ± 1.34 (SE = 0.08)	−0.6 ± 1.11	0

				*TZDs versus SUs*					

(i) Charbonnel et al. [24] Pioglitazone versus Gliclazide	317	8.71 ± 1.00	NA	−0.89 ± 1.29 (SE = 0.07272)	313	8.53 ± 0.89	NA	−0.77 ± 1.18 (SE = 0.06666)	−0.12
(ii) Garber et al. [25] Rosiglitazone versus Glibenclamide	152	8.43 ± 1.20	7.17 ± 1.43	−1.1 ± 1.30^a^	153	8.47 ± 1.25	6.70 ± 1.37	−1.5 ± 1.29^a^	0.4
(iii) Umpierrez et al. [26] Pioglitazone versus Glimepiride	107	8.31 ± 0.77	NA	−1.23 ± 0.76 (SE = 0.073)	96	8.40 ± 0.72	NA	−1.3 ± 0.75 (SE = 0.077)	0.07
(iv) Hamann et al. [27] Rosiglitazone versus Glibenclamide, Gliclazide	285	8.0 ± 0.9	NA	−0.78 ± 1.01 (SE = 0.06)	288	8.0 ± 1.0	NA	−0.86 ± 1.02 (SE = 0.06)	0.08

				*TZD versus TZD*					

(i) Derosa et al. [28] Pioglitazone versus Rosiglitazone	48	8.2 ± 0.8	6.8 ± 0.3	−1.4 ± 0.7^a^	48	8.1 ± 0.9	6.8 ± 0.5	−1.3 ± 0.78^a^	−0.1

				*Insulin versus SU*					

(i) Kvapil et al. [29] BIAsp 30 versus Glibenclamide	108	9.24 ± 1.32 (SE = 0.127)	7.52 ± 1.09 (SE = 0.105)	−1.72 ± 1.22^a^	114	9.45 ± 1.39 (SE = 0.130)	7.8 ± 1.25 (SE = 0.118)	−1.65 ± 1.33^a^	−0.07

Data are mean ± SD values. NA: not available. ^a^SD calculated from SD baseline and final values.

**Table 3:** Summary of FPG (mg/dL) between the treatment and the control groups.

Study	Treatment				Control				Difference
	*n*	Baseline	Final	Change	*n*	Baseline	Final	Change	between groups
				*TZDs versus DPP IV inhs.*					

(i) Scott et al. [22] Rosiglitazone versus Sitagliptin	87	156.9 ± 31.6	132.8 ± 29.9	−24.5 ± 33.55	92	157.2 ± 30.7	145.8 ± 35.3	−11.7 ± 33.52	−12.8
(ii) Bolli et al. [23] Pioglitazone versus Vildagliptin	295	198.20 ± 48.65 (11.0 ± 2.7 mmol/L)	NA	−28.83 ± 48.95 (−1.6 mmol/L)	280	198.40 ± 46.85 (11.3 ± 2.6 mmol/L)	NA	−18.02 ± 49.22 (−1.0 mmol/L)	−10.81

				*TZDs versus SUs*					

(i) Charbonnel et al. [24] Pioglitazone versus Gliclazide	317	212.61 ± 55.86 (11.8 ± 3.1 mmol/L)	NA	−32.43 ± 59.28 (−1.8 ± 0.18 (SE) mmol/L)	313	203.60 ± 46.85 (11.3 ± 2.6 mmol/L)	NA	−19.82 ± 57.30 (−1.1 ± 0.18 (SE) mmol/L)	−12.61
(ii) Garber et al. [25] Rosiglitazone versus Glibenclamide	152	188.95 ± 36.32	151.05 ± 43.16	−36 ± 40.18^a^	153	193.68 ± 34.21	143.27 ± 46.16	−46 ± 41.50^a^	10
(iii) Umpierrez et al. [26] Pioglitazone versus Glimepiride	107	184.2 ± 42.14	NA	−39.7 ± 35.38 (SE = 3.42)	96	180.4 ± 38.72	NA	−34.1 ± 35.57 (SE = 3.63)	−5.6
(iv) Hamann et al. [27] Rosiglitazone versus Glibenclamide, Gliclazide	285	189.19 ± 50.45 (10.5 ± 2.8 mmol/L)	NA	−41.26 ± 48.67 (−2.29 ± 0.16 (SE) mmol/L)	288	183.78 ± 52.25 (10.2 ± 2.9 mmol/L)	NA	−40.54 ± 48.92 (−2.25 ± 0.16 (SE) mmol/L)	−0.72

				*TZD versus TZD*					

(i) Derosa et al. [28] Pioglitazone versus Rosiglitazone	48	161 ± 24	140 ± 15	−21 ± 21^a^	48	164 ± 27	146 ± 18	−18 ± 23.81^a^	−3

Data are mean ± SD values. NA: not available. ^a^SD calculated from SD baseline and final values. To convert mg/dL to mmol/L multiply by 0.0555.

**Table 4:** Summary of FPI (*μ*U/mL) between the treatment and the control groups.

Study	Treatment				Control				Difference
	*n*	Baseline	Final	Change	*n*	Baseline	Final	Change	between groups
				*TZD versus DPP IV inh.*					

(i) Scott et al. [22] Rosiglitazone versus Sitagliptin	73	15.1 ± 9.6	11.2 ± 8.8	−3.7 ± 6.54	79	14.7 ± 9.9	14.5 ± 8.6	−0.2 ± 6.35	−3.5

				*TZDs versus SUs*					

(i) Charbonnel et al. [24] Pioglitazone versus Gliclazide	317	15.3 ± 11.70	NA	−2.61 ± 10.48^b^ (−18.1 pmol/L)	313	15.0 ± 10.07	NA	1.90 ± 10.48^b^ (13.2 pmol/L)	−4.51
(ii) Garber et al. [25] Rosiglitazone versus Glibenclamide	152	18 ± 11	NA	−7 ± 10.48^b^	153	15 ± 11	NA	−3 ± 10.48^b^	−4
(iii) Umpierrez et al. [26] Pioglitazone versus Glimepiride	107	16.9 ± 15.2	NA	−5.18 ± 11.90 (SE = 1.15)	96	14.8 ± 8.95	NA	6.21 ± 11.95 (SE = 1.22)	−11.39
(iv) Hamann et al. [27] Rosiglitazone versus Glibenclamide, Gliclazide	236	12.40 ± 10.56 (86.12 ± 73.34 pmol/L)	8.26 ± 5.78 (57.36 ± 40.13 pmol/L)	−4.14 ± 9.16^a^	229	11.83 ± 11.38 (82.15 ± 79.04 pmol/L)	11.95 ± 8.95 (82.98 ± 62.15 pmol/L)	0.12 ± 10.38^a^	−4.26

				*TZD versus TZD*					

(i) Derosa et al. [28] Pioglitazone versus Rosiglitazone	48	25.5 ± 6.1	20.2 ± 4.9	−5.3 ± 5.60^a^	48	26.1 ± 5.9	22.2 ± 5.2	−3.9 ± 5.58^a^	1.4

Data are mean ± SD values. NA: not available. ^a^SD calculated from SD baseline and final values. ^b^SD calculated from pooled standard deviation of Umpierrez et al. [26] and Hamann et al. [27]. To convert *μ*U/mL to pmol/L multiply by 6.945.
